# PEA-OXA Mitigates Oxaliplatin-Induced Painful Neuropathy through NF-κB/Nrf-2 Axis

**DOI:** 10.3390/ijms22083927

**Published:** 2021-04-10

**Authors:** Michela Campolo, Marika Lanza, Irene Paterniti, Alessia Filippone, Alessio Ardizzone, Giovanna Casili, Sarah A. Scuderi, Caterina Puglisi, Marzia Mare, Lorenzo Memeo, Salvatore Cuzzocrea, Emanuela Esposito

**Affiliations:** 1 Department of Chemical, Biological, Pharmaceutical and Environmental Sciences, University of Messina, Viale Ferdinando Stagno D’Alcontres, 98166 Messina, Italy; campolom@unime.it (M.C.); lanzam@unime.it (M.L.); ipaterniti@unime.it (I.P.); alessia.filippone@unime.it (A.F.); aleardizzone@unime.it (A.A.); gcasili@unime.it (G.C.); sarahadriana.scuderi@unime.it (S.A.S.); salvator@unime.it (S.C.); 2 IOM Ricerca Srl, Via Penninazzo 11, 95029 Viagrande, Italy; caterina.puglisi@grupposamed.com; 3 Istituto Oncologico Del Mediterraneo Spa, Via Penninazzo 7, 95029 Viagrande, Italy; marzia.mare@grupposamed.com (M.M.); lorenzo.memeo@grupposamed.com (L.M.)

**Keywords:** palmitoylethanolamide ultramicronized (PEAum), 2-pentadecyl-2-oxazoline (PEA-OXA), oxaliplatin (L-OHP), pain, dorsal root ganglia (DRG)

## Abstract

Chemotherapy-induced neuropathy is a common, dose-dependent adverse effect of several antineoplastics, such as oxaliplatin (L-OHP). The aim of the present work was to evaluate the potential beneficial effects of 2-pentadecyl-2-oxazoline (PEA-OXA) in a murine model of oxaliplatin-induced peripheral neuropathy (OIPN). OIPN was induced by an intraperitoneally injection of L-OHP in rats on five consecutive days (D0–4) for a final cumulative dose of 10 mg/kg. PEA-OXA and ultramicronized palmitoylethanolamide (PEAum), both 10 mg/kg, were given orally 15–20 min prior (L-OHP) and sacrifice was made on day 25. Our results demonstrated that PEA-OXA, more than PEAum, reduced the development of hypersensitivity in rats; this was associated with the reduction in hyperactivation of glia cells and the increased production of proinflammatory cytokines in the dorsal horn of the spinal cord, accompanied by an upregulation of neurotrophic factors in the dorsal root ganglia (DRG). Moreover, we showed that PEA-OXA reduced L-OHP damage via a reduction in NF-κB pathway activation and a modulation of Nrf-2 pathways. Our findings identify PEA-OXA as a therapeutic target in chemotherapy-induced painful neuropathy, through the biomolecular signaling NF-κB/Nrf-2 axis, thanks to its abilities to counteract L-OHP damage. Therefore, we can consider PEA-OXA as a promising adjunct to chemotherapy to reduce chronic pain in patients.

## 1. Introduction

Chemotherapy-induced peripheral neuropathy (CIPN) is a frequent complication of common anticancer therapies [1]. CIPN represents a debilitating side-effect arising from the administration of chemotherapeutic agents and its incidence is growing exponentially [2]. The chemotherapy drugs that can origin CIPN include vinca alkaloids, taxanes, and, above all, platinum analogues. In particular, CIPN develops during platinum drug treatment and the symptoms may persist for 2–6 months after cessation of chemotherapy [3]. Oxaliplatin-induced peripheral neuropathy (OIPN) initiates through the accumulation of platinum adducts in dorsal root ganglion (DRG) and trigeminal ganglion (TG) neurons, leading to a modification of axon function, as well as myelin sheath, neuronal cell body, and glial structures [3]. The pathological mechanisms underlying CIPN are still unclear; however, a considerable involvement of oxidative stress and inflammation has been recently recognized, leading to an increase in nociceptive trigeminal ganglion excitability. Recent reports demonstrated the protective ability of the endogenous fatty acid amide palmitoylethanolamide (PEA) in the rat model of paclitaxel-induced allodynia [4]. PEA belongs to the family of endogenous lipid mediators of *N*-acylethanolamines (NAEs), and it has been shown to play very important roles in the control of inflammation and in analgesic phenomena [5]; this increases its potential as a new therapeutic approach, while also considering its good safety profile and lack of toxicity and genotoxic potential in both rodent and human studies [6,7].

However, the administration of PEA undergoes problems related to its metabolism; therefore, it was considered more useful to administer it in association with oxazoline, forming the 2-pentadecyl-2-oxazoline of palmitoylethanolamide (PEA-OXA) [8]. In fact, among the enzymes that degrade NAEs, *N*-acylethanolamine acid amidase (NAAA) is specific for PEA [9]; therefore, the pharmacological inhibition, by oxazoline, of this enzyme can increase PEA bioavailability, improving its activity [8]. This hypothesis was confirmed in many papers, showing an improved PEA-OXA effectiveness compared to ultramicronized PEA (PEAum) alone in reducing inflammation and hyperalgesia [10,11]. On this basis, the aim of this study was to evaluate the neuroprotective and analgesic effects of PEA-OXA on neuropathic pain induced by oxaliplatin (L-OHP) treatment.

## 2. Results

### 2.1. PEA-OXA Prevented Mechano-Allodynia and Thermal Hyperalgesia and Suppressed OIPN Symptoms in an Operant-Based Mechanical Conflict System (MCS)

**Figure 1:**
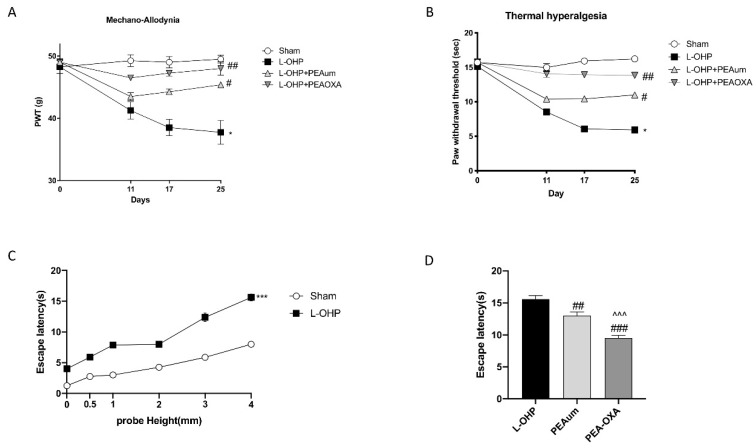
Effect of 2-pentadecyl-2-oxazoline (PEA-OXA) on allodynia and hyperalgesia. When compared to day 0, administration of oxaliplatin (L-OHP) but not saline resulted in a time-dependent development of mechano-allodynia (**A**) and mechano-hyperalgesia (**B**). The development of mechano-hypersensitivity was blocked by daily oral injections (Days 0–4) of PEA-OXA (10 mg/kg/day), more significative then ultramicronized palmitoylethanolamide (PEAum) (10 mg/kg). The *Y*-axis corresponding to paw withdrawal threshold (PWT) is cropped for clarity. (**C**) Time course of escape latencies of chemotherapy-induced peripheral neuropathy (CIPN) and control rats measured with 3 mm probe height after weekly injections of L-OHP or saline. (**D**) Oral administration of PEA-OXA at 10 mg/kg, more than PEAum, significantly reduced escape latencies when injected with L-OHP. Data are representative of at least three independent experiments. Values are means ± standard error of the mean (SEM) of 20 animals for each group. * *p* < 0.05 vs. sham; # *p* < 0.05 vs. L-OHP; ## *p* < 0.01 vs. L-OHP; ### *p* < 0.001 vs. L-OHP; ^^^ *p* < 0.001 vs. PEAum; *** *p* < 0.001.

The five day (D) consecutive treatment with L-OHP (D0–4) produced a time-dependent development of mechano-allodynia and thermal-hyperalgesia, as evidenced by a significant reduction in the pain thresholds, evidenced by paw withdrawal threshold (PWT) of the rats subject to mechanical and thermal stimulation. The stimulus was evident starting from D11 (beginning) and reaching a peak at D17 which continued until the end of our observation period (D25; times related to the first injection) (Figure 1A,B). The pretreatment with PEA-OXA, much more than PEAum, mitigated mechano-allodynia and thermal hyperalgesia as of D11, by preventing the onset of pain (Figure 1A,B). On day 25, an operant-based MCS was assessed (Figure 1C,D). Latency to escape the light chamber increased as probe height increased in both sham and L-OHP rats suggesting that elevated probes are relatively more nociceptive. A comparison between groups across all probe heights showed that L-OHP rats exhibited a significantly higher mean escape latency compared to the sham group (Figure 1C). The effect of systemic administration of PEAum and PEA-OXA on escape latency was assessed in rats following MCS training. Tests conducted in each compound group, prior to drug administration, confirmed that MCS escape latencies were consistent with those observed in L-OHP-treated rats in the stimulus response assessment for the 0 and 3 mm probe conditions. Mean escape latency at 3 mm was significantly reduced with PEA-OXA at doses of 10 mg/kg, much more than PEAum when compared to vehicle (L-OHP group) (Figure 1D).

### 2.2. PEA-OXA Preserved Neuronal Morphological Change and Sciatic Nerve Damage Following L-OHP Injection

**Figure 2:**
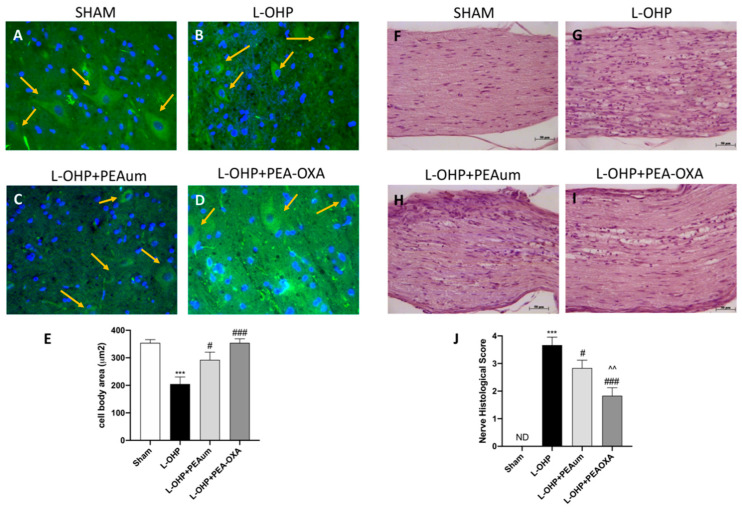
Effect of PEA-OXA on neuronal and peripheral nerve morphology. The immunofluorescence staining for β-III tubulin (green) and 4′,6′-diamidino-2-phenylindole (DAPI) (blue) was made on lumbar spinal cord. L-OHP injection changed neuronal morphology (**B**,**E**), compared to sham group (**A**,**E**), while PEA-OXA significantly preserved neuronal shape (**D**,**E**) much more then PEAum (**C**,**E**). Scale bar = 20 μm (particle). Hematoxylin and eosin (H&E) was made on sciatic nerve 25 days following L-OHP injection. PEA-OXA significantly preserved sciatic nerve tissue architecture degeneration (**I**,**J)**, more than PEAum (**H**,**J**), compared to L-OHP group (**G**,**J**). Data are representative of at least three independent experiments. Values are means ± SEM of 20 animals for each group. One-way ANOVA test; *** *p* < 0.001 vs. sham; # *p* < 0.05 vs. L-OHP; ### *p* < 0.001 vs. L-OHP; ^^ *p* < 0.01 vs. PEAum.

To investigate the “state of health” of neurons following L-OHP treatment, the lumbar section of the spinal cord was stained with β-III tubulin as a neuronal marker [12,13]. In our data, the treatment with L-OHP did not reduce the number of neurons, but visibly changed neuronal morphology, making the neurons much smaller (Figure 2B,E) compared to the sham group (Figure 2A,E). PEAum treatment partially reversed this modification (Figure 2C,E) that was definitively protected by PEA-OXA pretreatment (Figure 2D,E).

Histological analysis by staining with hematoxylin and eosin (H&E) showed different areas of edema with abundant presence of infiltrated and degraded myelin layers at 25 days in the sciatic nerve of mice treated with L-OHP (Figure 2G,J) compare to the sham group (Figure 2F,J). The treatment with PEA-OXA significantly preserved tissue architecture (Figure 2I,J) more effectively than the PEAum (Figure 2H,J).

### 2.3. Effect of PEA-OXA on Nuclear Factor Kappa-Light-Chain-Enhancer of Activated B Cells (NF-κB) Pathway and Proinflammatory Cytokines Induced by L-OHP Injection

**Figure 3:**
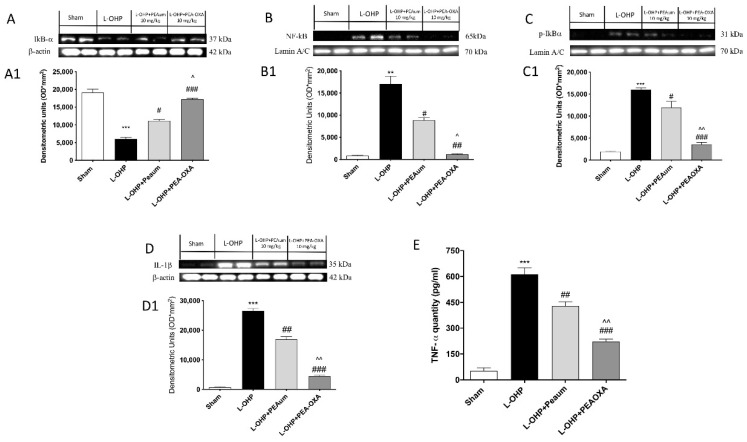
Effect of PEA-OXA on NF-κB pathway and proinflammatory cytokines. The expression of IκB-α, NF-κB, p-IκB-α, and IL-1β was observed by Western blot analysis, in lumbar spinal cord, 25 days after L-OHP injections. We observed that L-OHP injections induced a decrease in IκB-α expression compared to sham group (**A**,**A1**), which was markedly reduced following PEA-OXA treatment (**A**,**A1**). Moreover, a significant NF-κB nuclear translocation was seen following L-OHP injection, which was almost completely inhibited by PEA-OXA 10 mg/kg treatment, much more then PEAum (**B**,**B1**), as well as IκB-α phosphorylation (**C**,**C1**). Furthermore, PEA-OXA treatment, compared to PEAum, markedly reduced both IL-1β expression (**D**) and TNF-α levels (**E**). Data are means ± SEM of 10 mice for each group. One-way ANOVA test. A representative blot of lysates obtained from each group is shown and densitometry analysis of all animals is reported (*n* = 10 mice from each group). *** *p* < 0.001 vs. sham; ** *p* < 0.01 vs. sham; # *p*< 0.05 vs. L-OHP; ## *p*< 0.01 vs. L-OHP; ### *p* < 0.001 vs. L-OHP; ^ *p* < 0.05 vs. PEAum; ^^ *p* < 0.01 vs. PEAum.

The obtained results showed that L-OHP induced a significant increase in the nuclear translocation of the NF-κB p65 protein in the lumbar spinal tissue compared to the control animals (Figure 3B, see densitometric analysis 3B1). The treatment with PEA-OXA in L-OHP-injected animals significantly impeded the translocation of NF-κB p65 (Figure 3B, see densitometric analysis 3B1) and nuclear factor of kappa light polypeptide gene enhancer in B-cells inhibitor (IκB)-α phosphorylation (Figure 3C, see densitometric analysis 3C1). On the contrary, the degradation of IκB-α was significantly increased in the lumbar portion of the spinal cord of animals with L-OHP, whereas it was partially restored with PEA-OXA treatment (Figure 3A, see densitometric analysis 3A1). The production of cytokine interleukin (IL)-1β significantly increased following L-OHP injection (Figure 3D, see densitometric analysis 3D1); the treatment with PEA-OXA significantly reduced IL-1β expression, much more than PEAum (Figure 3D, see densitometric analysis 3D1). Moreover, we evaluated the levels of the cytokine tumor necrosis factor (TNF)-α using an ELISA kit. L-OHP injection induced a large increase in TNF-α expression compared to the control group; treatment with PEA-OXA, even more than treatment with PEAum, reduced the expression of this proinflammatory cytokine (Figure 3E).

### 2.4. PEA-OXA Treatment Stimulated Antioxidant Response in the Lumbar Spinal Cord

**Figure 4:**
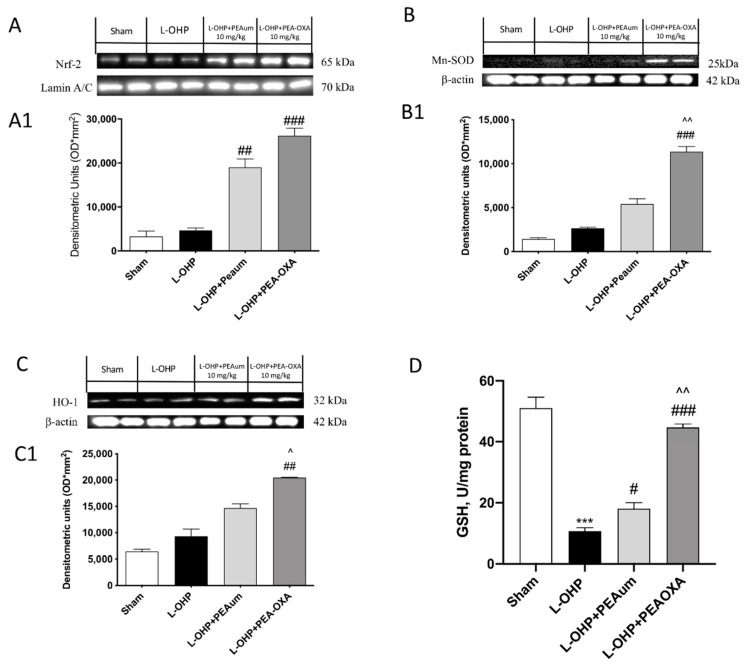
Effect of PEA-OXA on Nrf-2, Mn-superoxide dismutase (MnSOD), and HO-1 expression and reduced glutathione (GSH) levels. The expression of Nrf-2, Mn-SOD, and HO-1 was observed by Western blot analysis in lumbar spinal cord. Nuclear Nrf-2 expression increased following L-OHP injection, compared to sham group (**A**), while PEA-OXA administration, 10 mg/kg, upregulated Nrf-2 activity (**A**). The same result was obtained for MnSOD (**B**) and HO-1 expression, which was notably increased following L-OHP compared to sham groups (**C**), while PEA-OXA treatment significantly upregulated Mn-SOD and HO-1 expression, much more then PEAum (**B**,**C**). L-OHP decreased GSH levels compared to control group, while PEA-OXA treatment restored GSH levels, much more than PEAum (**D**). Data are means ± SEM of 10 mice for each group. A representative blot of lysates obtained from each group is shown and densitometry analysis of all animals is reported (*n* = 10 mice from each group). One-way ANOVA test; *** *p* < 0.001 vs. sham; # *p* < 0.05 vs. L-OHP; ## *p* < 0.01 vs. L-OHP; ### *p* < 0.001 vs. L-OHP; ^ *p* < 0.05 vs. PEAum; ^^ *p* < 0.01 vs. PEAum.

We evaluated the effect of PEA-OXA on the nuclear factor erythroid 2-related factor (Nrf)-2 pathway, a regulator of cellular resistance to oxidants, by Western blot analysis. Nrf-2 expression was not stimulated following administration of L-OHP as compared with control rats (Figure 4A, see densitometric analysis 4A1). PEA-OXA treatment significantly upregulated Nrf-2 levels much more than PEAum (Figure 4A, see densitometric analysis 4A1). Moreover, in L-OHP-injected rats, there was no significative modification of levels of Heme Oxygenase (HO)-1 or Mn-superoxide dismutase (MnSOD) (Figure 4B,C respectively, see densitometric analysis 4(B1,C1) respectively) compared to sham group.

Our data showed a very slight increase in Mn-SOD in L-OHP group, compared to the sham group, highlighting a physiological antioxidant response that contrasts L-OHP intoxication. This increase would be attributable to the direct effect that cytokines (such as IL-1β and TNF-α) exert on dismutase expression following L-OHP injection.

Interestingly, treatment with 10 mg/kg PEA-OXA strongly increased HO-1 and Mn-SOD expression (Figure 4B,C respectively, see densitometric analysis Figure 4(B1,C1) respectively), whereas, in L-OHP rats treated with 10 mg/kg of PEAum, the antioxidant enzymes activities increased partially (Figure 4B,C respectively, see densitometric analysis Figure 4(B1,C1) respectively). Furthermore, administration of PEA-OXA 10 mg/kg, more than PEAum, had the ability to restore reduced glutathione (GSH) levels close to basal levels.

### 2.5. PEA-OXA Reduced Astrogliosis and IL-17 Production in the Lumbar Spinal Cord

**Figure 5:**
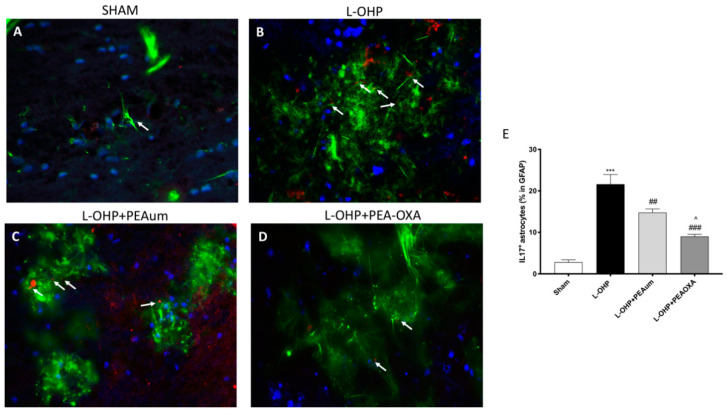
Effect of PEA-OXA on GFAP and IL-17 expression. The immunofluorescence staining for GFAP (green) and IL-17 (red) was made in the lumbar spinal cord sections. Considerable astrogliosis with production of IL17 was present in L-OHP panels (**B**,**E**), compared to the sham group (**A**,**E**). The treatment with PEA-OXA did not show any significant immunoreactivity of IL-17 in astrocytes (**D**,**E**), much more then PEAum (**C**,**E**). Scale bar = 20 μm (particle). Data are means ± SEM from *n* = 10 mice/group. Counting of colocalized cells confirmed our data (**E**). The colocalization image was analyzed with image J software. *** *p* < 0.001 vs. sham; ## *p* < 0.01 and ### *p* < 0.001 vs. L-OHP; ^ *p* < 0.05 vs. PEAum.

We analyzed with immunofluorescence staining the expression of IL-17 in astrocytes, evaluated through Glial fibrillary acidic protein (GFAP). In this study, we demonstrated that L-OHP induced a prominent astrogliosis with the consequent production of IL-17 (Figure 5B,E), compared to sham animals (Figure 5A,E). Instead, treatment with PEAum reduced the number of reactive astrocytes (Figure 5C,E), which was almost completely reversed by PEA-OXA (Figure 5D,E).

### 2.6. PEA-OXA Stimulates Growth Factor Expression in the DRG

**Figure 6:**
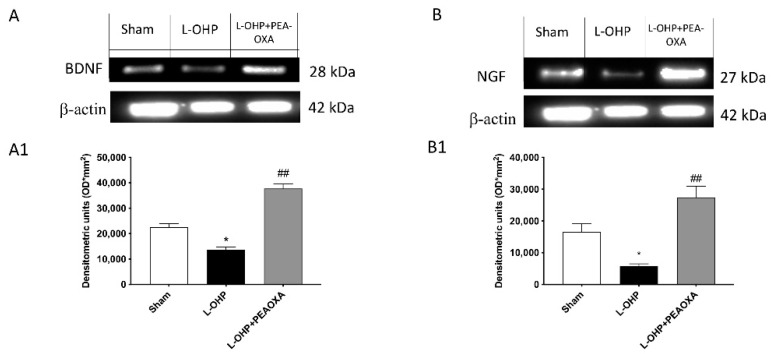
Effect of PEA-OXA on BDNF and NGF expression. Western blot analysis for BDNF and NGF was made in the dorsal root ganglia (DRG). A significant reduction in neurotrophic factor, BDNF, and NGF was seen following L-OHP injection compared to the sham group (**A**,**B** respectively), while PEA-OXA considerably upregulated BDNF and NGF expression (**A**,**B**); see densitometric analysis (**A1**,**B1**). The DRG samples were pulled for each group. Data are means ± SEM of 10 mice for each group. A representative blot of lysates obtained from each group is shown and densitometry analysis of all animals is reported (*n* = 10 mice from each group). One-way ANOVA test; * *p* < 0.05 vs. sham; ## *p* < 0.01 vs. L-OHP.

To investigate whether PEA-OXA modulates the pain process through regulation of neurotrophic factor levels, DRG samples were studied through Western blot analysis. Unpredictably, the treatment with PEA-OXA stimulated the release of both brain-derived neurotrophic factor (BDNF) and nerve growth factor (NGF), suggesting a role in the promotion of growth and differentiation of novel neurons and synapses (Figure 6A,B, see densitometric analysis Figure 6(A1,B1)).

## 3. Discussion

Neuropathic pain is among the most severe side-effects during tumor chemotherapy. OIPN is characterized by a lowered mechanical and thermal nociceptive threshold [14], where mitochondrial dysfunction, oxidative stress, and inflammation cover a key role [15,16,17]. Numerous studies have been directed to identify molecules with the capacity to relieve the symptoms of OIPN; on this basis, different endogenous lipid mediators have been identified as capable of reducing the inflammatory processes caused by OIPN [18]. PEA plays an important role among the lipid mediators in neuropathic pain [10,19,20], as well as in controlling of inflammatory phenomena and oxidative stress [7,21]. Recently, it was found that the pharmacological inhibition of the enzyme NAAA produces a marked analgesic and anti-inflammatory effect [9,22]; therefore, treatment with PEA-OXA associates the known effects of PEA with those of NAAA inhibition, which has, among other effects, an increase in endogenous endocannabinoid levels. To support these data, different studies confirmed the neuroprotective and anti-inflammatory effects of PEA-OXA in animal models of central nervous system (CNS) trauma and neurodegenerative diseases [8,11,23,24]. PEA-OXA exerts a neuroprotective effect through a reduction in astrocyte activation and an increase in the release of neurotrophic factors [8]. The anti-inflammatory effects of PEA-OXA have been shown to be related to inhibition of the NF-κB pathway and upregulation of the Nrf-2 pathway [8].

As with cancer patients, this murine model shows a delay in the onset of mechano-hypersensitivity that mimics the clinical phenomenon observed with patients [25].

More specifically, CIPN represented principally a sensory neuropathy [26]; sensory symptoms usually develop in a typical distribution called “glove and stocking” neuropathy, denoting the first involvement of the distal part of hand and feet. Symptoms include altered touch sensation or paresthesia, as well as painful sensations and mechanical or thermal allodynia or hyperalgesia [27]. Actually, the position of PEA in the modulation of pain has been well studied as a clinical strategy, as an alternative to the traditional drugs such as opioids and analgesics for neuropathic pain [7]. Moreover, it has been shown that the pharmacological inhibition of NAAA enhanced the analgesic effect of PEA [28]. Our results confirmed that L-OHP injury significantly promoted in a time-dependent manner the development of neuropathic pain, developing mechano-allodynia and thermal hyperalgesia. However, the treatment with PEA-OXA significantly prevented the evolution of pain, highlighting its analgesic effect.

The onset of CIPN, occurring independently of chemotherapy agent mechanisms, is associated with neuronal morphological changes ascribable to microtubule organization of cytoskeleton [29]. On this basis, we looked to the β-III tubulin isotype known to confer dynamic assets to microtubules, which represents an important biomarker for resistance to microtubule-targeting chemotherapeutics in breast and other types of solid cancer [30]. Lenn et al. highlighted a correlation between the altered function of peripheral neurons after L-OHP and their structural changes in DRG cell bodies and axons of the sciatic nerve, observing a high incidence of multinucleolated cell bodies with eccentric nucleoli [31]. L-OHP injury provoked changes to the neuronal shape and size, as demonstrated by Wafai et al., on myenteric neurons, underlying a significant reduction in neuronal area in mice subjected to repeated L-OHP injections [32]. The mechanism proposed via which CNS neurons would lose their morphology following L-OHP administration is related to an alteration of the tight junction (TJ) proteins zonula occludens-1 (ZO-1) and F-actin, thus highlighting blood–brain barrier (BBB) alteration. Moreover, the chemotherapy-dependent BBB alterations were supported by an evidenced ROS formation and activation of the ER stress/ATP release signaling pathway. Cleary, in our results, neurons subjected to L-OHP treatment changed their shape, becoming much smaller than the control neurons. The pretreatment with PEA-OXA surprisingly preserved neuronal morphology, as well as tissue damage caused by L-OHP in the sciatic nerve.

Functional impairment in neurons, due to chemotherapeutic agents, occurs due to inflammation, oxidative stress, apoptosis, and electrophysiological disturbances that are triggered following the treatment [33].

Neuroinflammation (increased glial activation and proinflammatory cytokine production) in the spinal cord contributes significantly to the development of central sensitization associated with pain from different etiologies, including pain induced by L-OHP [34]. In fact, it has been well studied how neuroinflammation represents an event that often occurs during CIPN in the spinal cord. Moreover, it has been reported that the development of neuropathic pain induced by platinum compounds is linked with an increased formation of cytokines in the spinal cord, demonstrating the involvement of the proinflammatory signaling pathway driven by NF-κB [25,34]. Specifically, as highlighted by several studies [35,36], TNF-αis a cytokine associated with neuropathy, and its inhibition could be a promising target for neuropathic patients [37].

Our results clearly evidenced the activation of the NF-κB signaling pathway following treatment. The pretreatment with PEA-OXA significantly attenuated the nuclear translocation of the subunit p65 and the consequent production of proinflammatory cytokines such as IL-1β and TNF-α.

The increase in neuroinflammation is in concomitance with oxidative stress, already identified to be responsible for neuronal damage in different models of neuropathies [11,38,39,40]. Therefore, it is established that oxidative stress contributes to the pathophysiology of CIPN [33]. In particular, chemotherapy induced mitochondrial dysfunction and corresponding oxidative stress generation, mediating the peripheral nerve damage [41,42,43]. However, physiologically, the redox imbalance produced in neuronal cells could be modulated through adjustment of Nrf-2 [44]; therefore, we looked at the influence of PEA-OXA on erythroid factor activation. Moreover, there exists cross-talk between activation of the Nrf-2 system and anti-inflammatory effects via interactions with NF-κB, through identification of NF-κB binding sites in the promoter region of the Nrf-2 gene [45,46]; this cross-talk showed good efficacy in many models of peripheral neuropathy [47,48].

Our data visibly revealed an upregulation of Nrf-2 and, consequently, the transcription of antioxidant enzymes such as MnSOD and HO-1 following PEA-OXA treatment.

In the context of oxidative stress referred to OIPN, it emerged that GSH also plays a key role in counteracting nerve damage [33]. Preclinical and clinical data [49,50] confirmed its promising role in preventing OIPN, without reducing the pharmacological activity of oxaliplatin [49]. Our results indicate that PEA-OXA treatment considerably restored GSH levels reduced by OHP injection, thus suggesting that PEA-OXA is a good enhancer of antioxidant activity and a good adjuvant in maintaining cellular redox homeostasis.

Evidence suggests that CIPN is associated with activation of spinal astrocytes [34]. Many papers showed that L-OHP treatment induced mechanical hypersensitivity, which coincided with hyperactivation of astrocytes and the consequent upregulation of proinflammatory cytokines [39]. Recently, IL-17 was found to modulate inflammatory responses associated with neuropathic pain [51]. Moreover, IL-17 can also be produced by spinal cord astrocytes and astrocytic IL-17 may play a role in inflammatory pain [52]; in fact, in animal models, it has been shown that the overexpression of IL-17 in spinal astrocytes is adequate to induce mechanical allodynia following chemotherapy as reported by Luo et al. [53]. On this basis, we looked at IL-17 production by astrocytes, which was considerably increased following OXA treatment. However, PEA-OXA was able to preserve GFAP/IL-17 hyperexpression in the spinal cord.

The involvement of neurotrophic factors in the developmental mechanism of CIPN has not yet been well identified and still remains controversial; despite this, reduced levels of the BDNF family member NGF were found to be associated with CIPN development in patients with cervical carcinoma [54]. The involvement of NGF in the pathogenesis of the OIPN has been reported in several experimental studies, demonstrating NGF neuroprotective properties in in vitro, in vivo, and clinical studies [55,56].

In addition, a prospective study on patients treated with cisplatin and paclitaxel evidenced a significant correlation between the severity of the peripheral neurotoxicity and the circulating levels of NGF [54]; in particular, a decrease in NGF circulating levels was observed during cisplatin and L-OHP administration in rats closely correlated with the onset of peripheral neuropathy [57]. Many scientific articles [58,59] underlined BDNF as a prognostic biomarker in predicting CIPN in cancer patients.

Consistent with the above, in the dorsal root ganglia, we found a significant reduction in NGF and BDNF expression 25 days after L-OHP injection, while the treatment with PEA-OXA significantly upregulated the expression of NGF when compared to the vehicle group. This represents a key step in the neuroprotective effect of PEA-OXA, which may have a role in the promotion of growth and differentiation of novel neurons and synapses. However, given the conflicting views on the role of neurotrophins in CIPN, further preclinical and clinical investigations are needed to provide more insight into the function of these important factors.

## 4. Material and Methods

### 4.1. Materials

L-OHP and the other compounds were acquired from Sigma-Aldrich Company Ltd. (Milan, Italy). All other chemicals were of the highest viable grade available. All stock solutions were arranged in nonpyrogenic saline (0.9% NaCl; Baxter, Milan, Italy). PEA and PEA-OXA were provided by the Epitech Group (Saccolongo, Padua, Italy).

### 4.2. Animals

Adult male Wistar rats (Envigo, Milan, Italy), weight 200–250 g, were employed. Animals were divided into groups of three with free access to water and food under standardized humidity and temperature. This study was approved by the University of Messina Review Board for the care of animals (Protocol number 8/U-apr16). Animal care was in conformity with current legislation for the protection of animals used for scientific purposes (Directive 2010/63/EU, 9 April 2016) and the ARRIVE guidelines.

### 4.3. OIPN Model

L-OHP or its vehicle (5% dextrose) was injected intraperitoneally (i.p.) in rats on five consecutive days (D0–4) at an injection volume of 0.2 mL for a final cumulative dose of 10 mg/kg at the end of the 5 days. This low-dose paradigm does not trigger kidney injury, as reported by Bennett et al. [40].

#### Experimental Groups

(1) Sham + vehicle (veh): rats received saline orally (*n* = 20);
(2) Sham + PEAum: rats received PEAum (10 mg/kg) orally (*n* = 20);
(3) Sham + PEA-OXA: rats received PEA-OXA (10 mg/kg) orally (*n* = 20);
(4) L-OHP: rats received L-OHP (10 mg/kg) intraperitoneally (*n* = 20);
(5) L-OHP + PEAum: rats received PEAum orally at the dose of 10 mg/kg 20 min before OXA injection (*n* = 20);
(6) L-OHP + PEA-OXA: rats received PEA-OXA orally at the dose of 10 mg/kg 20 min before OXA injection (*n* = 20).

The dose and the animal model were based on previous in vivo studies [10,38,39,60]. Experimental data regarding groups 2 and 3 are not shown because PEAum and PEA-OXA administration resulted in neither toxicity nor an improvement in comparison to sham control.

### 4.4. Synthesis of PEA and PEA-OXA

PEA and PEA-OXA were synthesized as previously described by Impellizzeri et al. [8].

### 4.5. Behavioral Tests

Behavioral measurements to assess mechano-hypersensitivity were always taken prior to the administration of test substances (D0) and on day 7 (D7), day11 (D11), and day 25 (D25). Data were coded and then given to two blinded observers independently for scoring. Two mice were filmed in two separate fields at once. They were assigned randomly to collecting data so that all combinations of groups were present.

Mechano-allodynia was measured as previously described [25,39]. Briefly, rats were allowed to acclimate to behavioral chambers for 15 min prior to measuring the mechanical paw withdrawal thresholds (PWT, (g)) using an electronic Von Frey test (dynamic plantar aesthesiometer, model 37450; Ugo Basile, Italy) with the cutoff set at 50 g. Mechano-allodynia was defined by a significant (*p* < 0.05) reduction in mechanical mean absolute PWT (g) at forces that failed to elicit withdrawal responses before chemotherapy treatment (D0). Thermal hyperalgesia was assessed in rat hind paws using a digital hot-plate apparatus (Harvard apparatus, USA). The hot plate was thermostatically maintained at 52.0 ± 0.2 °C and the escape phenomenon was measured in seconds as jumping or hind paw lifting/licking. A cutoff time limit of 40 s was selected in order to avoid tissue injury. Each response latency was quantified as the paw withdrawal latency (PWL) [61]. Animals receiving chemotherapeutic agents in the presence or absence of the experimental test substances tested did not display any signs of toxicity, i.e., they exhibited normal posture, grooming, locomotor behavior, normal hair coat, no signs of piloerection or ocular porphyrin discharge, and normal body weight gain comparable to vehicle-treated rats.

#### Mechanical Conflict System (MCS) for Drug Assessment

A mechanical conflict system (MCS) (Stoelting, Wood Dale, IL, US) was used to assess the operant behavior of OIPN and control rats as described by Harte et al. (Harte et al., 2014). The apparatus is made of bright and dark compartments separated by a 15 inch (38 cm) long middle compartment arranged with an array of height-adjustable probes (0.5–4.0 mm). Before baseline measurements, four training sessions were carried out over 4 days. On the day of baseline recordings, rats were tested for escape latencies with nociceptive probes set at variable heights (0.5, 1.0, 2.0, 3.0, and 4.0 mm). Each compound was tested with the 3 mm probe condition as the standard noxious test stimulus because the stimulus response assessment indicated that escape latency at the 3 mm condition was significantly higher than at the 0 mm (no probe) condition. For each trial, rats were placed individually into the light compartment with the light turned off and the escape door closed. Following 10 s of dark acclimatization, the compartment light was turned on for the rest of the trial. The escape door was released 20 s after the light was turned on. The animal was considered inside the dark compartment when all four paws were placed completely inside that compartment. For rats that initiated walking on the nail bed but failed to reach the dark compartment during the 3 min testing period, a cutoff latency of 120 s was used. The operant test was assessed on the 25th day. All trials were video-recorded for offline analysis.

### 4.6. Histology

At the end of the 25 days, standard hematoxylin and eosin staining (H&E) was performed for histological examination as previously described [10]. The portions of sciatic nerve tissue were included in paraffin, and then cut in sections with a thickness of 7 μm. They were then deparaffinized with xylene and stained with H&E staining. The sciatic nerve tissue samples were then observed by light microscopy (Leica QWin V3, Cambridge, UK). To quantify infiltration in the sciatic nerve, scores of 0, 1, 2, 3, and 4 were used, indicating 0%, 1–25%, 26–50%, 51–75%, and >75% infiltration, respectively [62].

### 4.7. Immunofluorescence of β-III Tubulin, GFAP, and IL-17

Spinal cord tissue sections of 7 μm were incubated with one of the following primary antibodies: anti-IL17 rabbit polyclonal (1:100; sc-374218, Santa Cruz Biotechnology, CA, USA) or mouse anti-GFAP (1:100; sc-33673, Santa Cruz Biotechnology, CA, USA) and anti- β-III tubulin (1:100; sc-80005, Santa Cruz Biotechnology, CA, USA), in a humidified chamber at 37 °C overnight. Sections were washed with phosphate buffered saline (PBS) and were incubated with secondary antibody TEXAS RED-conjugated anti-rabbit Alexa Fluor-594 antibody (1:1000 in PBS, *v*/*v*, Molecular Probes, Altrincham, UK) and with fluorescein isothiocyanate (FITC)-conjugated anti-mouse Alexa Fluor-488 antibody (1:2000 *v*/*v*, Molecular Probes, Altrincham, UK) for 1 h at 37 °C. Sections were washed and, for nuclear staining, 4′,6′-diamidino-2-phenylindole (DAPI; Hoechst, Frankfurt, Germany) (2 μg/mL) in PBS was added. Sections were observed and photographed at  100× magnification using a Leica DM2000 microscope. All analyses were carried out by two observers blinded to the treatment. For immunofluorescence, a 40× magnification is shown (20 µm scale bar).

For morphological analysis of β-III tubulin-positive cells, two random images were acquired from each section. The cell body was the criterion for selection. The cell processes were completely within the field of view, and the body of an individual cell was distinct from neighboring cells.

Cell body area was measured using Image J Version 8.2.1 software (US National Institutes of Health, Bethesda, MD, USA, https://imagej.nih.gov/ij/, accessed on 9 April 2021).

### 4.8. Western Blot Analysis for IκB-α, NF-κB, p-IκB α IL-1β, HO-1, MnSOD, Nrf-2, BDNF, and NGF

Western blots were done as previously described [23]. Cytosolic and nuclear fractions of lumbar spinal cord and DRG tissues were prepared and used for the detection of protein expressions. The membranes were blocked with 1× PBS and 5% (*w*/*v*) non-fat dried milk (PM) for 40 min at room temperature; membranes from spinal cord homogenates were probed with one of the following primary antibodies: anti-heme oxygenase (HO)-1 (1:500; sc-390991 Santa Cruz Biotechnology, CA, USA), anti-MnSOD (1:500; sc-137254, Santa Cruz Biotechnology, CA, USA), anti-IκB-α (1:500; sc-1643, Santa Cruz Biotechnology, CA, USA), and anti-IL-1β (1:500, sc-52013, Santa Cruz Biotechnology, CA, USA) in the cytosolic fraction, and anti-NF-κB (1:500; sc-390991, Santa Cruz Biotechnology, CA, USA), anti-Nrf2 (1:500; sc-13032, Santa Cruz Biotechnology, CA, USA), and anti-phosphorylated-IκB-α in the nuclear fraction, in 1× PBS, 0.1% Tween-20, and 5% *w*/*v* non-fat dried milk (PMT) at 4 °C overnight. Membranes from DRGs were incubated with BDNF (1:500; sc-20981, Santa Cruz Biotechnology, CA, USA) and NGF (1:500; sc-365944, Santa Cruz Biotechnology, CA, USA). Membranes were incubated with peroxidase-conjugated bovine anti-mouse immunoglobulin G (IgG) secondary antibody or peroxidase-conjugated goat antirabbit IgG (1:2000, Jackson ImmunoResearch, West Grove, PA, USA) for 1 h at room temperature. Blots were also incubated with a primary antibody against β-actin protein (1:10,000; Sigma-Aldrich Corp., St. Louis, MO, USA) or lamin A/C (1:10,000; Sigma-Aldrich Corp, St. Louis, MO, USA), which were used as internal standards. The relative expressions of the protein bands of MnSOD (21.6 kDa), HO-1 (33 kDa), IκB-α (37 kDa), NF-κB p65 (65 kDa), IL-1β (35 kDa), Nrf2 (61 kDa), and p-IκB-α (31 kDa), were detected in the lumbar spinal cord. BDNF (28 kDa) and NGF (27 kDa) were detected in the DRG. In the experiments, including the Western blot analysis, a representative blot is displayed and a densitometric analysis is provided in each figure. All analyses were carried out by two observers blinded to the treatment.

### 4.9. DRG Dissection

The spinal vertebrae were exposed by rapid midline dissection and lateral reflection of overlying skin and muscle. The lumbar spinal cord tissue was obtained after dorsal laminectomy. Tissue was dissected on ice-cooled glass into dorsal and ventral aspects, using the central spinal canal.

As reference, dorsal root ganglia were obtained from lumbar segments after removal of the spinal cord. All tissues were immediately frozen on dry ice.

### 4.10. ELISA Kit for TNF-α

ELISA assay was performed as previously described by Casili et al. [63].

Lumbar spinal cord tissues were homogenized with lysis buffer (Pierce #87787, Thermo Fisher Scientific, Waltham, MA, USA) and then supplemented with a protease inhibitor cocktail (Sigma-Aldrich, Rehovot, Israel). Successively, samples were homogenized and centrifuged at 14,000× *g* for 10 min at 4 °C; supernatants were stored at −20 °C. TNF-α levels was measured by ELISA assay, according to the manufacturer’s instructions.

### 4.11. Measurement of GSH

GSH levels was assessed as previously described by Fusco et al. [64].

In lumbar spinal cord tissues, GSH levels were quantified through activity assay kits (Nanjing Jiancheng Bioengineering Institute).

### 4.12. Statistical Analysis

All values, in the figures, were evaluated as means ± SEM. The results were examined by one-way ANOVA followed by a Bonferroni post hoc test for multiple comparisons and Student’s *t*-test to compare two groups (unpaired test); *p*-values < 0.05 were considered statistically significant.

## 5. Conclusions

Overall, our results showed that the treatment with PEA-OXA at a dose of 10 mg/kg daily, given before L-OHP injection, was able to reduce the development of inflammatory and oxidative processes by modulation of the NF-κB/Nrf-2 axis, preserving neuronal morphology and active reparative processes via neurotrophin upregulation. Much more significant, the treatment with PEA-OXA had a marked analgesic effect, inhibiting the mechanical allodynia and thermal hyperalgesia. However further studies are necessary to evidence other mechanisms induced by L-OHP; moreover, there are limitations and challenges with animal models of neuropathy because clinical models are essential for a better understanding of the behavioral consequences, as well as the numerous complex histopathological cascades and inflammatory signaling pathways.

With these results, we can suggest that our compound offers a potential benefit by mitigating CINP, allowing the enhancement of chemotherapy treatment when given in adjunct with PEA-OXA.

## 6. Patents

Salvatore Cuzzocrea is the coinventor of patent WO2013/121449 A8 (Epitech Group SpA). Methods for the modulation of amidases capable of hydrolyzing *N*-acylethanolamines useable in the therapy of inflammatory diseases. Moreover, Dr Cuzzocrea is also a coinventor with the Epitech group of the following patents: 1. EP 2814489; 2. EP 2821083; 3. EP 2985037; 4. 102015000067344.

## Data Availability

The data presented in this study are available on request from the corresponding author.
